# Maternal Right Ventricular and Left Atrial Function in Uncomplicated Twin Pregnancies: A Longitudinal Study

**DOI:** 10.3390/jcm11185432

**Published:** 2022-09-15

**Authors:** Rossana Orabona, Edoardo Sciatti, Enrico Vizzardi, Ivano Bonadei, Marco Metra, Enrico Sartori, Tiziana Frusca, Antonio Pinna, Rino Bellocco, Federico Prefumo

**Affiliations:** 1 Maternal Fetal Medicine Unit, Department of Obstetrics and Gynecology, University of Brescia, 25123 Brescia, Italy; 2 Section of Cardiovascular Diseases, Department of Medical and Surgical Specialties, Radiological Sciences and Public Health, University of Brescia, 25123 Brescia, Italy; 3 Department of Obstetrics and Gynecology, University of Parma, 43121 Parma, Italy; 4 Department of Statistics and Quantitative Methods, University of Milano-Bicocca, 20126 Milan, Italy; 5 Department of Medical Epidemiology and Biostatistics, Karolinska Institutet, 171 65 Stockholm, Sweden

**Keywords:** twin pregnancy, echocardiography, diastole, systole, 2d strain, speckle-tracking echocardiography, right ventricular, left atrial, tissue doppler imaging

## Abstract

Objective: The knowledge regarding maternal cardiovascular hemodynamic adaptation in twin pregnancies is incomplete. We performed a longitudinal investigation of maternal right ventricular (RV) and left atrial (LA) function in a cohort of uncomplicated twin pregnancies compared to singleton pregnancies. Study design: Healthy women with uncomplicated twin pregnancies were prospectively enrolled and assessed by transthoracic echocardiography at 10–15 weeks’ (w) gestation (T1), 19-26 w gestation (T2), and 30–38 w gestation (T3). Subjects with uneventful singleton pregnancies were selected as controls at the same gestational ages. Cardiac findings were compared to those of women with uneventful singleton gestations. RV systolic and diastolic functions were assessed by conventional echocardiography (FAC, TAPSE, sPAP, E, A, DT) and tissue Doppler imaging (TDI) (E’, A’, S’, IVA, IVCT, IVRT, ET, MPI), and LA dimensions were calculated. Speckle-tracking imaging was also applied to evaluate RV global longitudinal strain and LA 2D strains (at LV end-systole (LAS) and at atrial contraction (LAA)). Results: Overall, 30 uncomplicated twin and 30 uncomplicated singleton pregnancies were included. Regarding maternal RV function in twins, all the parameters (FAC, TAPSE, sPAP, E, A, E/A, DT, E/E’, IVA, IVCT, MPI and 2D longitudinal strain) were almost stable throughout gestation, with the exception of the TDI findings (E’ decreased from T1 to T3 (*p* = 0.03), while E’/A’ increased from T1 to T2 and then decreased (*p* = 0.01); A’ and basal S’ increased (*p* = 0.04 and *p* = 0.03, respectively), while IVRT and ET significantly decreased (*p* = 0.009 and *p* = 0.007, respectively)). These findings were similar to those found for singleton pregnancies. LA dimensions significantly increased throughout gestation in both twins and singletons (*p* < 0.001), without intergroup difference. LA strains did not vary during either twin or singleton pregnancies, except for LAA in T1, which was higher among twins than among singletons. Conclusion: Maternal RV and LA function in uncomplicated twin pregnancies does not seem to undergo more significant changes than in singletons, being characterized by similar findings in RV systolic and diastolic functions, as well as LA dimensions and strains.

## 1. Introduction

Physiological changes in maternal hemodynamics are required for cardiovascular (CV) adaptation to pregnancy [1], in order to meet the increased metabolic demands of both the mother and the baby. Systemic vascular resistance and blood pressure decrease from the first trimester, with a nadir in the second trimester, while cardiac output increases until delivery. Consequently, cardiac remodeling occurs to face these changes, principally by means of increased LVM [2,3]. Furthermore, a failure in these modifications during pregnancy may result in a rise in feto-maternal morbidity. Although it can be hypothesized that this phenomenon in women with twin pregnancies might be responsible for the higher risk of adverse pregnancy outcomes in such cases, a paucity of data is available on maternal hemodynamics in twins [4,5,6,7]. With twin pregnancy being acknowledged by the American Society for Reproductive Medicine as carrying high risk [8], maternal hemodynamics in women with twin pregnancies are thought to show greater modifications than in singletons. Some of these changes have previously been reported in the literature. In a large cross-sectional study, Kametas et al. documented more pronounced hemodynamic changes in twins than in singletons, including a rise in cardiac output, left ventricular mass, and ejection fraction of the left ventricle [4]. In addition to left ventricular assessment, understanding maternal CV adaptation to the pregnant state requires the analysis of the impact of pregnancy on right ventricular (RV) and left atrial (LA) performance. To the best of our knowledge, there are no data focusing on RV and LA findings throughout gestation. Furthermore, the use of relatively new diagnostic strategies, such as speckle-tracking echocardiography (STE), may have potential benefits with respect to additional risk stratification as a result of improved delineation of cardiac performance status. STE promises to reduce inter- and intraobserver variability in the assessment of myocardial function and to improve healthcare cost effectiveness through the early identification of subclinical disease [9]. The main purpose of this study was to longitudinally evaluate RV and LA function by means of conventional echocardiography, tissue Doppler imaging, and STE in a population of uncomplicated twin pregnancies compared to singleton gestations in order to evaluate whether twins imply more pronounced changes in maternal RV and LA findings than singletons do.

## 2. Materials and Methods

As described in a previous paper on the same population [10], from February 2015 to September 2016, all patients with viable twin pregnancy undergoing sonographic first-trimester screening at the Maternal Fetal Medicine Unit of the Obstetrics and Gynecology Department of the University of Brescia, Italy for the 1st trimester exam were enrolled in a prospective and consecutive way. Increased nuchal translucency, abnormal sonographic findings of either fetus, and monoamnionicity represented a priori exclusion criteria. Subjects were scheduled for a combined assessment including fetal sonographic evaluation and maternal cardiologic assessment (i.e., blood pressure measurement and echocardiography) in a stable-temperature environment thrice during pregnancy (T1, 10–15 weeks’ gestation (w); T2, 19–26 w; T3, 30–38 w). Every woman signed their informed consent. The study complied with the Declaration of Helsinki, was approved by the local ethics committee, and was run following the STROBE indications [11]. Exclusion criteria were: at least one complication in a precedent pregnancy (e.g., pre-eclampsia, fetal growth restriction (FGR), intrauterine fetal death, three or more consecutive spontaneous miscarriages); ascertained or suspected fetal anomalies in the present pregnancy; FGR of one or both fetuses (defined as an estimated fetal weight of either fetus < 5th centile, or discrepancy between estimated fetal weight greater than 20%); amniotic fluid imbalance or suspected twin-to-twin transfusion syndrome; maternal history of chronic diseases (e.g., hypertensive disorders, diabetes mellitus, renal or immune disorders); traditional CV risk factors (e.g., smoking habit, dyslipidemia, obesity); drug use. According to the same inclusion criteria, we enrolled healthy subjects with singleton pregnancies attending our Unit during the same span of time. Data about chorionicity were based upon the first-trimester report. Demographic and clinical data were collected from obstetrical charts for every woman. The exams were performed by a physician blinded to the women’s data to reduce intra- and interobserver variability. Thirty-nine subjects with uncomplicated twin pregnancies and 34 with healthy singleton ones fitted the inclusion criteria and were enrolled in a prospective way [10]. Among them, 13 women were then excluded (9 twin and 4 singleton), so that 30 women per group reached the end of the study (the causes for leaving the study are listed in Table S1).

### 2.1. Blood Pressure Measurement

A standard, calibrated, electronic sphygmomanometer (OMRON Healthcare, Hoofddorp, The Netherlands) was used to measure blood pressure, at rest, at a 45° sitting angle. Systolic blood pressure (SBP) is elevated when higher than 140 mmHg and diastolic blood pressure (DBP) if greater than 90 mmHg. Two more measurements were taken at the arm with the highest blood pressure, and the average value was calculated. Blood pressure was assessed by the same staff member, during the same part of the day, and adopting the same device. Mean arterial pressure (MAP) was defined as (SBP + 2 × DBP)/3.

### 2.2. Conventional and Tissue Doppler Echocardiography

Echocardiographic examinations were performed using the Vivid 7 machine (GE Healthcare, Milwaukee, WI, USA) with a 3.5 MHz transducer. Digital loops were stored on the hard disk of the echocardiograph for on-line and off-line analyses and transferred to a workstation EchoPac, Vingmed (GE Healthcare, Milwaukee, WI, USA) for off-line analysis. Participants were studied in the left lateral decubitus position, and images were acquired from standard parasternal and apical windows. RV and LA dimensions were obtained according to current guidelines [12]. RV systolic function was evaluated according to the guidelines, calculating fractional area change (FAC), tricuspid annular plane systolic excursion (TAPSE), basal S’ wave, and isovolumic acceleration (IVA) at TDI [13]. RV diastolic function was defined according to guidelines, considering transtricuspid Doppler inflows and TDI at the lateral basal segment [13]. Myocardial performance index (MPI) was calculated as (IVCT + IVRT)/ET, where IVCT is isovolumic contraction time, IVRT is isovolumic relaxation time, and ET is ejection time at TDI [13]. Valvular alteration was screened according to the guidelines [14,15]. Systolic pulmonary artery pressure (sPAP) was obtained by adding the right atrial pressure estimate to Bernoulli’s simplified equation for tricuspid regurgitation jet velocity by means of continuous wave Doppler [13].

### 2.3. Speckle-Tracking Echocardiography

Two-dimensional (2D) strain calculates myocardial deformation from a 2D point of view. Negative strain means shortening, while positive indicates thickening of a given myocardial segment. STE analysis using the commercially available automated function image technique was applied for the assessment of RA and LA longitudinal strain from an apical four-chamber view [16]. The endocardial borders were traced in the end-systolic frame of the 2D images. Speckles were tracked frame by frame through the RV and LA walls until the software automatically approved the tracking of the six segments. Segments that failed to track were manually adjusted by the operator until the software approved them. For the right ventricle, RV GLS was calculated. In addition, we studied left atrial (LA) 2D strain, calculating its longitudinal peak at the end of LV systole (LA_S_) and at atrial contraction (LA_A_) in apical four-chamber view. Moreover, we measured the time to peak longitudinal strain (TPLS) from the R wave of QRS to LA_S_. Figures describing this technique have been reported elsewhere [17]. Finally, we derived atrial stiffness by dividing LV E/E’ to LA_S_ (using average septal and lateral E’), as previously described [17,18,19]. We defined images as being of good quality when at least 4 segments out of 6 did not require manual interpolation. No patients were excluded from STE analyses.

### 2.4. Statistical Analysis

Continuous variables are reported as mean ± standard deviation (SD), and categorical ones as frequency (*n*) and percentage (%). Student’s *t*-test was run to compare the means for continuous variables. The *χ*^2^ test (or Fisher’s exact test) was adopted to assess differences between proportions.

Every clinical outcome was studied by means of multilevel mixed-effects linear regression analysis. This strategy is highly appropriate for longitudinal data: with respect to a standard two-way ANOVA for repeated measurements, it considers each outcome as a linear model with random intercepts and slopes. This assumption makes it possible to fit models with a general covariance matrix depending on time and also makes it possible to use all nonmissing information on repeated data. The model to be fitted was as follows:

(1) $$Y_{ij}=\beta_{0}+\beta_{01}*Twin+\beta_{1}t_{j}+\beta_{2}t_{j}^{2}+b_{i0}+b_{i1}t_{j}+e_{ij}$$

where:

*i* represents the subject, *j* the time of measurement.

$\beta_{0}$ is the fixed intercept.

$\beta_{01}$ is the parameter related to the twin dependency.

$\beta_{1},\beta_{2}$ represent the type of time trend (linear or quadratic).

$b_{i0}+b_{i1}t_{ij}+e_{ij}$ is the error term expressed in terms of random intercept, random slope, and residuals, where terms $b_{i0}$, $\;b_{i1}$, and $e_{ij}$ have zero mean.

Finally, a quantile regression analysis was performed to design the boundary curves. This analysis extends the longitudinal mixed model one, focusing on the chosen quantile. 

To better compare the models, they were corrected for age.

For every outcome, the best-fitted model was selected by means of the minimum Akaike criterion. To discover the best model, an initial linear model hypothesis, adopting the twin pregnancy as covariate, was analyzed, and if needed, its polynomial degree was elevated.

R and R-Studio were used to analyze databases and to run traditional statistics (mean, SD, etc.), while longitudinal data analysis was performed using Stata 14.2 with xtmixed() function, and Quantile Regression was performed by the lqmm() method using the lqmm R package [20].

For every statistic, *p*-value < 0.05 was regarded as significant.

## 3. Results

Eleven out of the 30 cases constituted monochorionic pregnancy (37%). Three visits (one per trimester) were attended by 24/30 (80%) twins and 28/30 (93%) singletons. In the twins group, 2/30 (7%) women missed the T2 visit, while 4/30 (13%) twins and 2/30 (7%) singletons delayed the T3 visit.

The demographic characteristics of the cohort were described in a previous paper [10]. Age was higher in women with twin pregnancy (34.5 ± 4.3 vs 31.5 ± 4.0; *p* < 0.01), while delivery time was earlier (35^+3^ ± 2^+2^ vs 39^+2^ ± 2^+4^; *p* < 0.001) and the Caesarean section rate was higher (87% vs. 27%; *p* < 0.001). Body mass index and body surface area increased during pregnancy in both groups, with a significant quadratic trend (<0.00009 for all), but without intergroup differences.

**Figure 1:**
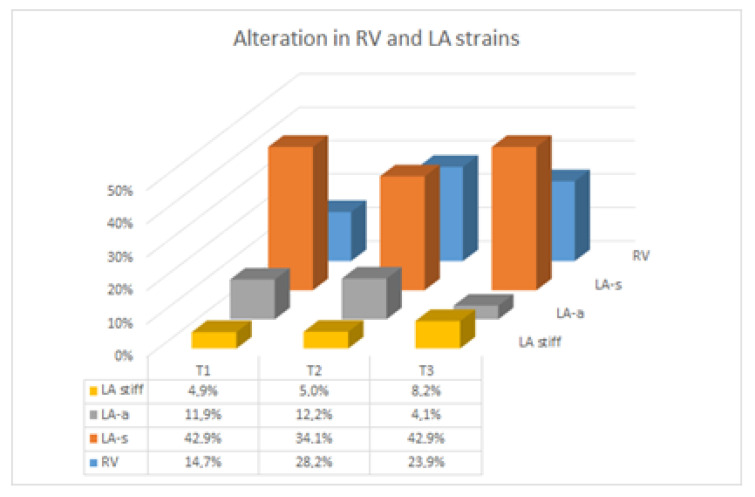
Right ventricular (RV) and left atrial (LA) 2D strain alterations in the whole study cohort.

Details regarding hemodynamic parameters were reported in a previous paper that studied the same population [8]. In both groups, blood pressure dropped from T1 to T2 and then rose to T3, and no cases of hypertension disorders of pregnancy were found, even thought blood pressure was slightly higher in the twin group across all three trimesters [10]. Data regarding the right ventricle are shown in Table 1. The majority of RV variables (e.g., FAC, TAPSE, sPAP, E, A, E/A, DT, E/E’, IVA, IVCT, MPI and 2D longitudinal strain) were almost stable throughout twin gestation, and seemed to be substantially similar to those found during singleton gestation (except for a tendency for E and E/A to be lower in twins, although this was not confirmed on the basis of longitudinal analysis in all in which $\beta_{1}$ was significant and the twin pregnancy $\beta_{01}$ was not significant). TAPSE and basal IVA showed a significant increasing trend throughout singleton pregnancy, while basal S’ at T2 was greater among twins than in singletons. In fact, the longitudinal analysis shows that $\beta_{1}$ is significant in basal S’, but for IVA there are different slopes in singleton and twin pregnancy because $\beta_{01}$ is significant; in addition, there is a significant interaction factor between time and twin pregnancy that explains what was reported at T2. Diastolic function significantly changed throughout twin gestation. In particular, E’ decreased from T1 to T3 (*p* = 0.03), E’/A’ increased from T1 to T2 and then decreased (*p* = 0.01), A’ increased (*p* = 0.04) from T1 to T3, and IVRT and ET significantly decreased from T1 to T3 (*p* = 0.009 and *p* = 0.007, respectively). These findings were similar to those observed during singleton pregnancy, as also confirmed by the longitudinal model in which only $\beta_{1}$ was significant. Regarding the right ventricle, STE data were available for 34/60 (56.7%) subjects at T1, 39/58 (67.2%) at T2, and 46/54 (85.2%) at T3. Longitudinal RV 2D strain did not change across the trimesters, with no intergroup difference. It was altered in 5/34 cases (14.7%) at T1, 11/39 (28.2%) at T2 and 11/46 (23.9%) at T3, independently of multiple pregnancy or gestational age (Figure 1).

Data about the left atrium are listed in Table 2. LA dimensions significantly increased from T1 to T3, both in twins and singletons (<0.001), as confirmed by the longitudinal model in which only $\beta_{1}$ was significant. With respect to the LA STE data, they were available for 42/60 (70.0%) women at T1, 41/58 (70.7%) at T2 and 49/54 (90.7%) at T3, while LA stiffness data were available for 41 out of 60 cases (68.3%) at T1, 40 out of 58 (69.0%) at T2, and 49 out of 54 (90.7%) at T3. LA strains and stiffness did not vary throughout twin or singleton gestation. LA_S_ strain was altered in 18/42 (42.9%) women at T1, 14/41 (34.1%) at T2 and 21/49 (42.9%) at T3; LA_A_ strain was altered in 5/42 (11.9%) at T1, 5/41 (12.2%) at T2 and 2/49 (4.1%) at T3; LA stiffness was altered in 2/41 (4.9%) at T1, 2/40 (5.0%) at T2 and 4/49 (8.2%) at T3.

Table 3 highlights the linear regression models considering pregnancy status (twins or singletons) and gestational age as independent variables. Only basal IVA seemed to be related to either twin gestation or gestational age, but the remaining parameters were dependent only on gestational age.

## 4. Discussion

The main findings of this paper are: (1) maternal RV systolic and diastolic functions are almost stable throughout twin gestation and seem to be similar to those observed in women carrying uncomplicated singleton pregnancies; (2) RV longitudinal strain is altered in 15–30% of women in both groups; (3) maternal LA dimensions similarly increase throughout twin and singleton pregnancies; (4) LA strains do not differ between twin and singleton pregnancies, except for LA_A_ at T1, which is greater among twins; they were globally altered in about half of the women across all three trimesters.

The maternal CV system undergoes significant changes during gestation, including an increase in cardiac output and a decrease in maternal systemic vascular resistance [1,2,3]. This physiological adaptation ensures adequate uteroplacental circulation for fetal growth and development, while maladaptation has been associated with a higher risk of adverse outcome. Understanding the normal CV changes occurring during pregnancy is essential not only for caring for patients with CV disease, but also for trying to anticipate the occurrence of complications in the pregnancies of subjects with no pre-pregnancy disorders [21,22,23]. This is the first longitudinal study in twins focusing on the right ventricle and the left atrium, which are directly involved in maternal hemodynamics. The emphasis in obstetrics recently shifted towards left ventricular physiology, overshadowing the study of these cardiac chambers. The importance of the assessment of maternal RV function has been recognized not only in heart failure and RV myocardial infarction, but also in other clinical scenarios such as congenital heart diseases and pulmonary hypertension. In fact, RV diastolic dysfunction increases right-sided pressures leading to fluid retention and representing an important predictor of exercise capacity and survival in subjects suffering from cardiopulmonary disease [24]. We performed a longitudinal assessment of maternal RV systolic and diastolic functions starting from early gestation, and found that they remained almost stable throughout twin gestation and did not differ from the RV function of women carrying singleton pregnancies. IVA is a parameter of RV contractile function, and it is independent from preload and afterload changes in a physiological range [25]. To the best of our knowledge, no previous reports in the literature have assessed IVA during pregnancy. Because of its prognostic usefulness [26], we evaluated IVA in our patients, finding that it was dependent not only on gestational age, but also on multiple pregnancy. Moreover, we also analyzed RV longitudinal strain for the first time in pregnancy. We think that its knowledge is of pivotal importance given the advantages it brings [9]. Indeed, STE has been applied to overcome the limitations of conventional echocardiography. It is angle-independent, being not greatly influenced by preload or afterload and being not affected by heart movements [9]. Importantly, we found that 15−30% of women in both groups showed an altered RV longitudinal strain across all three trimesters, indicating the profound hemodynamic changes faced by the heart during pregnancy. Because of the increasing numbers of women with congenital heart disease hoping for a baby, it is important for clinicians dealing with such cases to be aware of the risk factors and presentation of cardiac conditions in pregnancy. For this reason, the assessment of RV function may be a crucial component in early pregnancy counseling, especially in women with congenital heart disease and pulmonary hypertension. 

With respect to the left atrium, it is well known that its principal role is to modulate CV performance by functioning as a reservoir for pulmonary venous return during ventricular systole, as a conduit for pulmonary venous return during early ventricular diastole, and as a booster pump for augmenting ventricular filling during late ventricular diastole [27]. Hunter et al. described a significantly larger increase in LA diameter in twin pregnancies than in singleton ones consistent with volume overload [28]. Conversely, in our cohort, the LA dimensions increased throughout twin pregnancy, with no significant differences being observed between twins and singletons. The most likely explanation of these differences in measurements between their report and ours is the small number of patients included in our study, since LA enlargement reflects hypervolemia occurring during pregnancy. Moreover, modifications to maternal cardiac function as a function of chorionicity were recently investigated by our group in a multicenter study [29]. The data showed a significant decrease in cardiac output and an increase in total vascular resistance in monochorionic compared to dichorionic twins. Furthermore, during monochorionic pregnancies, LA enlargement could be less pronounced than in dichorionic pregnancies due to the lower circulating volume, and this issue may also explain why we did not observe any differences between twins and singletons. For the first time in the literature, we evaluated the left atrium in pregnancy focusing on LA strains, describing similar values between twin and singleton pregnancies, except for LA_A_ at T1, which is greater among twins. Similar to what happens to the RV strain, the LA strains are altered in about half of the women in both groups across all three trimesters, probably due to the hypervolemic status typical of pregnancy. The role of the left atrium is emerging, with LA size and function being considered as prognostic variables in CV risk stratification [27]. For this reason, its clinical value could be used as an additional tool in early pregnancy counseling of women carrying high-risk pregnancies.

The main strengths of the present paper lie in its prospective study design, incorporating a longitudinal assessment of maternal cardiac function from the first to the third trimester of gestation. Moreover, a total evaluation of maternal RV and LA functions by means of STE has never carried out before in twin pregnancies. A detailed assessment of maternal hemodynamics in twins might offer better insights into the process of maternal adaptation to twin pregnancies, contributing more deeply to our understanding of the pathophysiology of CV complications that occur more frequently during twin gestations [30]. 

We acknowledge the following limitations to the study. Firstly, we cannot provide accurate reference ranges for the variables due to the small number of enrolled women. Secondly, the absence of a post-delivery evaluation means that we are not able to show which variables normalize or the point in time at which they do. Thirdly, as already noted, a great number of monochorionic twins were enrolled; considering that CO is higher in dichorionic twins [29], this could somewhat explain why no statistically significnt difference was demonstrated between twin and singleton pregnancies.

## 5. Conclusions

In conclusion, maternal RV and LA function in uncomplicated twin pregnancies does not seem to change more significantly than in singletons, being characterized by similar findings with respect to RV systolic and diastolic function, as well as LA dimensions and strains. Specifically, a certain number of women demonstrated altered RV and LA strains as a consequence of hypervolemia. The number of women in which this persists remains to be clarified. It is conceivable that these findings may represent another possible explanation for the increased risk of feto-maternal morbidity associated with twin pregnancy. Larger—and clinical—studies are needed to enrich the findings first described here.

## Figures and Tables

**Table 1:** Right ventricular findings obtained by echocardiography at each trimester in twin versus singleton pregnancies.

	1st	2nd	3rd	*p* (Trend)		Longitudinal Model	Twin Dipendency
FAC (%)	44.6 ± 8.3	46.8 ± 8.8	40.5 ± 11.0	0.2467	*p*	no	
	46.9 ± 7.0	45.6 ± 6.9	44.0 ± 8.2	0.1589	Twin		
	0.2754	0.587	0.2329		Singleton		
TAPSE (mm)	25 ± 5	28 ± 4	26 ± 4	0.8534	*p*	no	
	25 ± 4	27 ± 5	27 ± 5	0.0285	Twin		
	0.8192	0.2204	0.1518		Singleton		
sPAP (mmHg)	21 ± 3	20 ± 2	21 ± 3	0.1486	*p*	no	
	21 ± 4	21 ± 3	23 ± 4	0.1596	Twin		
	0.7456	0.3882	0.4992		Singleton		
E (m/s)	0.57 ± 0.17	0.61± 0.21	0.53 ± 0.14	0.6226	*p*	no	
	0.66 ± 0.17	0.61 ± 0.10	0.60 ± 0.12	0.0974	Twin		
	0.07286	0.9182	0.07409		Singleton		
A (m/s)	0.38 ± 0.12	0.44± 0.24	0.34 ± 0.13	0.4962	*p*	no	
	0.40 ± 0.14	0.38 ± 0.09	0.38 ± 0.10	0.8756	Twin		
	0.5959	0.2234	0.1828		Singleton		
E/A	1.53 ± 0.29	1.47 ± 0.32	1.71 ± 0.73	0.3374	*p*	no	
	1.72 ± 0.42	1.68 ± 0.41	1.62 ± 0.37	0.3302	Twin		
	0.06618	0.05839	0.6102		Singleton		
DT (ms)	173 ± 29	172 ± 14	174 ± 21	0.9539	*p*	no	
	177 ± 21	176 ± 17	184 ± 25	0.2312	Twin		
	0.5353	0.3116	0.1246		Singleton		
E’ (m/s)	−0.178 ± 0.043	−0.191 ± 0.041	−0.158 ± 0.032	0.0028	*p*	yes, linear	no
	−0.186 ± 0.036	−0.185 ± 0.036	−0.162 ± 0.033	0.0033	Twin	Y=0.187+0.0105 t	
	0.4327	0.5361	0.7052		Singleton		
A’ (m/s)	−0.118 ± 0.073	−0.159 ± 0.046	−0.151 ± 0.056	0.0359	*p*	yes, linear	no
	−0.129 ± 0.060	−0.138 ± 0.037	−0.173 ± 0.061	0.0060	Twin	Y=−0.125+0.0205 t	
	0.5275	0.07303	0.1869		Singleton		
S’ basal (m/s)	0.154 ± 0.025	0.183 ± 0.034	0.167 ± 0.034	0.0338	*p*	yes, linear no	
	0.167 ± 0.026	0.164 ± 0.027	0.175 ± 0.032	0.0382	Twin	Y=0.163+0.061 t	
	0.0552	0.02291	0.3972		Singleton		
S’ midwall (m/s)	0.124 ± 0.025	0.129 ± 0.024	0.5058	0.124 ± 0.025	*p*	no	
	0.131 ± 0.029	0.128 ± 0.027	0.633	0.131 ± 0.029	Twin		
	0.123 ± 0.034	0.139 ± 0.051	0.1655	0.123 ± 0.034	Singleton		
S’ apical (m/s)	0.076 ± 0.020	0.089 ± 0.025	0.080 ± 0.025	0.3395	*p*	no	
	0.080 ± 0.023	0.080 ± 0.029	0.087 ± 0.033	0.1880	Twin		
	0.4946	0.2242	0.3754		Singleton		
E’/A’	1.16 ± 1.11	1.31 ± 0.47	1.18 ± 0.46	0.0111 (**)	*p*	no	
	1.31 ± 0.77	1.41 ± 0.36	1.04 ± 0.39	0.8307	Twin		
	0.5635	0.3619	0.2552		Singleton		
E/E’	3.4 ± 1.1	3.3 ± 1.2	3.3 ± 0.7	NA	*p*	no	
	3.6 ± 1.3	3.2 ± 1.3	3.9 ± 1.3	0.4203	Twin		
	0.4923	0.6703	0.08289		Singleton		
IVA (m/s^2^)	4.2 ± 1.6	4.1 ± 1.3	4.1 ± 1.6	0.7039	*p*	yes, linear	yes
	3.6 ± 0.8	4.2 ± 1.5	5.2 ± 2.6	0.0042	Twin	Y=3.55+0.67 Tw +0.78 t−0.85 t∗Tw	
	0.06295	0.8148	0.07895		Singleton		
IVCT (ms)	66 ± 17	61 ± 18	65 ± 20	0.7107	*p*	no	
	75 ± 20	65 ± 15	68 ± 19	0.1472	Twin		
	0.08391	0.368	0.5175		Singleton		
IVRT (ms)	54 ± 24	53 ± 25	44 ± 12	0.0088	*p*	yes, linear	yes
	57 ± 21	52 ± 17	46 ± 15	0.1010	Twin	Y=55.77−4.92 t	
	0.6104	0.9262	0.7177		Singleton		
ET (ms)	282 ± 21	275 ± 29	265 ± 31	0.0071	*p*	no	
	276 ± 31	271 ± 29	258 ± 31	0.0048	Twin		
	0.4529	0.6007	0.4357		Singleton		
MPI	0.43 ± 0.13	0.42 ± 0.16	0.42 ± 0.11	0.7731	*p*	no	
	0.48 ± 0.13	0.44 ± 0.12	0.45 ± 0.11	NA	Twin		
	0.1138	0.645	0.3105		Singleton		
Longitudinal 2D strain (%)	−23.3 ± 5.4	−20.1 ± 3.6	−22.8 ± 4.3	0.8905	*p*	no	
	−21.0 ± 3.1	−20.9 ± 2.7	−20.7 ± 3.9	0.7041	Twin		
	0.1495	0.4422	0.1074		Singleton		

Data are given as mean ± SD for each trimester. P (trend) refers to a longitudinal regression run on the subgroup of 30 patients. If reported, the model has a *p*-value < 0.05; for details, see Table 3. FAC, fractional area change; TAPSE, tricuspid annular plane systolic excursion; sPAP, systolic pulmonary artery pressure; E, early diastolic wave; A, atrial (late) diastolic wave; DT, deceleration time; E’, early diastolic tissue wave; A’, atrial (late) diastolic tissue wave; S’, systolic tissue wave; (**) The trend has expression: $Y_{ij}=\beta_{0}+\beta_{1}t_{j}+\beta_{2}t_{j}^{2}+b_{1}t_{j}+e_{ij}$; IVA, isovolumic acceleration; IVCT, isovolumic contraction time; IVRT, isovolumic contraction relaxation; ET, ejection time; MPI, myocardial performance index; 2D, two dimensional.

**Table 2:** Left atrial findings obtained by echocardiography during each trimester for twin versus singleton pregnancies.

	1st	2nd	3rd	*p* (Trend)		Longitudinal Model	Twin Dipendency
Antero-posterior diameter (mm)	31 ± 4	35 ± 3	38 ± 3	<0.00009	*p*	yes, linear	no
	32 ± 5	35 ± 4	37 ± 5	<0.00009	Twin	Y=32.02+8.86 t	
	0.3284	0.9875	0.4128		Singleton		
Area (cm^2^)	12.8 ± 2.8	15.5 ± 2.7	15.4 ± 3.3	0.0001	*p*	yes, linear	no
	13.8 ± 3.3	16.0 ± 3.0	17.1 ± 6.5	0.0018	Twin	Y=13.64+1.53 t	
	0.1999	0.473	0.2292		Singleton		
Volume (mL)	27 ± 9	34 ± 10	36 ± 9	<0.00009 (***)	*p*	yes, linear	no
	26 ± 9	36 ± 15	36 ± 11	<0.00009 (**)	Twin	Y=26.53+12.37 t	
	0.7512	0.6751	0.9893		Singleton		
Volume index (mL/m^2)^	15.7 ± 5.3	19.4 ± 5.8	19.2 ± 5.2	<0.00009 (**)	*p*	yes, linear	no
	14.7 ± 5.5	20.3 ± 7.8	19.9 ± 5.5	<0.00009 (**)	Twin	Y=15.19+6.88 t	
	0.4985	0.6336	0.6737		Singleton		
LA_S_ 2D strain (%)	36.5 ± 19.8	33.8 ± 12.7	32.1 ± 12.4	0.2073	*p*	no	
	30.8 ± 11.4	38.2 ± 15.5	32.0 ± 14.3	0.9814	Twin		
	0.2497	0.33	0.981		Singleton		
LA_A_ 2D strain (%)	12.2 ± 7.0	10.6 ± 4.7	12.4 ± 5.2	0.9402	*p*	no	
	8.6 ± 3.9	12.8 ± 7.6	11.4 ± 5.6	0.1429	Twin		
	0.04893	0.2808	0.523		Singleton		
Stiffness	0.18 ± 0.11	0.20 ± 0.10	0.21 ± 0.10	0.15653	Twin	no	
	0.19 ± 0.10	0.16 ± 0.11	0.22 ± 0.12	0.2857	Singleton		
	0.7445	0.1893	0.8271		*p*		

Data are given as mean ± SD for each trimester. P (trend) refers to a longitudinal regression run on the subgroup of 30 patients. If reported, the model has a *p*-value < 0.05; for details, see Table 3. (***) The trend has expression: $Y_{ij}=\beta_{0}+\beta_{2}t_{j}^{2}+b_{1}t_{j}+e_{ij}$; (**) The trend has expression: $Y_{ij}=\beta_{0}+\beta_{1}t_{j}+\beta_{2}t_{j}^{2}+b_{1}t_{j}+e_{ij}$; LA, left atrial; LA_S_, LA 2D strain at LV end-systole; LA_A_, LA 2D strain at atrial contraction. Data are given as mean ± SD.

**Table 3:** Longitudinal regression analysis model based on: $Y_{ij}=\beta_{0}+\beta_{01}*Twin+\beta_{i1}t_{ij}+\beta_{2}t_{ij}^{2}+b_{0i\;}+b_{i1}t_{ij}+e_{ij}$.

	Base Value		β (Twin)		β (Time)		β (Time^2^)		Model
	Coeff.	*p*	Coeff.	*p*	Coeff.	*p*	Coeff.	*p*	*p*
BMI	22.60 ± 0.46	0.00			0.15 ± 0.13	0.26	0.45 ± 0.04	<0.0009	<0.00009
BSA	1.69 ± 0.02	0.00			0.006 ± 0.004	0.123	0.013 ± 0.01	<0.0009	<0.00009
E’	−0.1871947 ± 0.0049126	0.00			0.0104935 ± 0.0032543	0.001			0.0013
A’	−0.1248308 ± 0.0076612	0.00			−0.0205315 ± 0.0059499	0.001			0.0006
S’ basal	0.1626717 ± 0.0031544	0.00			0.00608 ± 0.0023773	0.011			0.0105
IVA (**)	3.550077 ± 0.2406082	0.00	0.6719929 ± 0.3383214	0.047	0.7676086 ± 0.238221	0.001			0.0142
IVRT	55.77271 ± 2.688193	0.00			−4.921733 ± 1.708916	0.004			0.0040
ET	279.7668 ± 3.277997	0.00			−8.860039 ± 2.244868	<0.0009			0.0001
LA antero−posterior diameter	32.01954 ± 0.5196185	0.00			2.821833 ± 0.2300515	<0.0009			<0.00009
LA area	13.56488 ± 0.3788698	0.00			1.532933 ± 0.3241711	<0.0009			<0.00009
LA volume	26.53448 ± 1.316003	0.00			12.37174 ± 2.05069	<0.0009	−3.975581 ± 0.9950033	<0.0009	<0.00009
LA volume index	15.1887 ± 0.7612453	0.00			6.876214 ± 1.167011	<0.0009	−2.45863 ± 0.5667266	<0.0009	<0.00009

BMI, body mass index; BSA, body surface area; E’, early diastolic tissue wave; A’, atrial (late) diastolic tissue wave; S’, systolic tissue wave; IVA, isovolumic acceleration; DT, deceleration time; IVRT, isovolumic contraction relaxation; ET, ejection time; LA, left atrial. (**) Interaction present between pregnancy and time: −0.8501304 ± 0.339043 (*p* = 0.012).

## Data Availability

Data available upon request.

## Supplementary Materials

The following are available online at https://www.mdpi.com/article/10.3390/jcm11185432/s1, Table S1: Reasons to leave the study. The last visit attended is reported in brackets.
