# Supplementary material for: Maternal Right Ventricular and Left Atrial Function in Uncomplicated Twin Pregnancies: A Longitudinal Study
Source: J Clin Med. 2022 Sep 15;11(18):5432. doi: 10.3390/jcm11185432 (PMC9503833; doi:10.3390/jcm11185432)
Supplement: Supplementary file 1 [file jcm-11-05432-s001.zip › jcm-1885996-supplementary.pdf]

Table S1. Reasons to leave the study. The last visit attended is reported in brackets.

Twin Pregnancy (*n* = 9)	Singleton Pregnancy (n = 4)
3 fetal malformation/chromosomopathy (T1, T1, T1)	
2 fetal loss (T1, T2)	1 pre-eclampsia and IUGR (T2)
1 HELLP syndrome and fetal loss (T2)	3 lost at follow-up (T1, T1)
3 lost at follow-up (T1, T1, T1)	

HELLP, hemolysis elevated liver enzymes low platelet, IUGR, intrauterine growth restriction.
